# Hypoxia evokes a sequence of raphe-pontomedullary network operations for inspiratory drive amplification and gasping

**DOI:** 10.1152/jn.00032.2024

**Published:** 2024-09-11

**Authors:** Sarah C. Nuding, Lauren S. Segers, Kimberly E. Iceman, Russell O’Connor, Jay B. Dean, Pierina A. Valarezo, Dale Shuman, Irene C. Solomon, Donald C. Bolser, Kendall F. Morris, Bruce G. Lindsey

**Affiliations:** ^1^Department of Molecular Pharmacology and Physiology, Morsani College of Medicine, University of South Florida, Tampa, Florida, United States; ^2^Department of Physiology and Biophysics, Renaissance School of Medicine at Stony Brook University, Stony Brook, New York, United States; ^3^Department of Physiological Sciences, College of Veterinary Medicine, University of Florida, Gainesville, Florida, United States

**Keywords:** apnea, apneusis, autoresuscitation, inspiratory drive amplification

## Abstract

Hypoxia can trigger a sequence of breathing-related behaviors, from augmentation to apneusis to apnea and gasping. Gasping is an autoresuscitative behavior that, via large tidal volumes and altered intrathoracic pressure, can enhance coronary perfusion, carotid blood flow, and sympathetic activity, and thereby coordinate cardiac and respiratory functions. We tested the hypotheses that hypoxia-evoked gasps are amplified through a disinhibitory microcircuit within the inspiratory neuron chain and that this drive is distributed via an efference copy mechanism. This generates coordinated gasplike discharges concurrently in other circuits of the raphe-pontomedullary respiratory network. Data were obtained from six decerebrate, vagotomized, neuromuscularly blocked, and artificially ventilated adult cats. Arterial blood pressure, phrenic nerve activity, end-tidal CO_2_, and other parameters were monitored. Hypoxia was produced by ventilation with a gas mixture of 5% O_2_ in nitrogen. Neuron spike trains were recorded at multiple pontomedullary sites simultaneously and evaluated for firing rate modulations and short-timescale correlations indicative of functional connectivity. Experimental perturbations evoked reconfiguration of raphe-pontomedullary circuits during initial augmentation, apneusis and augmented bursts, apnea, and gasping. Functional connectivity, altered firing rates, efference copy of gasp drive, and coordinated incremental blood pressure increases support a distributed brain stem network model for amplification and broadcasting of inspiratory drive during autoresuscitative gasping. Gasping begins with a reduction in inhibition by expiratory neurons and an initial loss of inspiratory drive during hypoxic apnea and culminates in autoresuscitative efforts.

**NEW & NOTEWORTHY** Severe hypoxia evokes a sequence of breathing-related behaviors culminating in gasping. We report firing rate modulations and short-timescale correlations in spike trains recorded simultaneously in the raphe-pontomedullary respiratory network during hypoxia. Our findings support a disinhibitory microcircuit and a distributed efference copy mechanism for amplification of gasping. Coordinated increments in blood pressure lead to a model for autoresuscitative bootstrapping of peripheral chemoreceptor reflexes, breathing, and sympathetic activity, complementing and extending prior work.

## Graphical abstract

**Figure:**
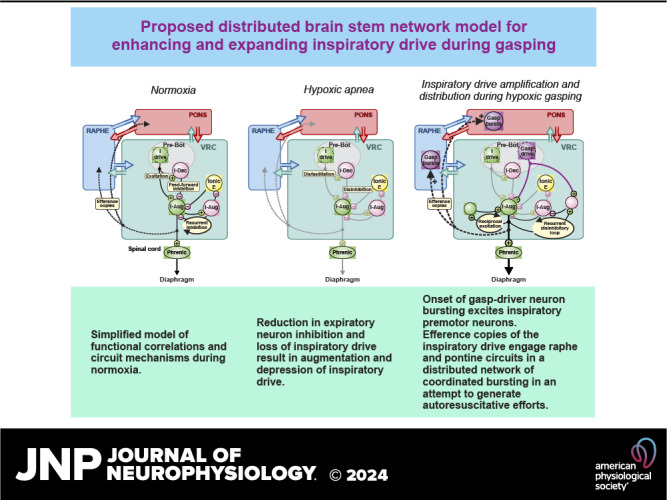

## INTRODUCTION

Hypoxia associated with a life-threatening event, such as cardiac arrest (1), a seizure (2), or an acute asthmatic episode (3), can trigger a sequence of breathing-related behaviors. An initial increase in breathing effort and frequency is driven by hypoxic stimulation of peripheral chemoreceptors (4). If this augmentation fails to resolve the hypoxia, the motor pattern transitions to apneusis, accompanied by augmented bursts (a.k.a. sighs) and respiratory depression (5, 6). An ensuing apnea or a dissolution of the respiratory rhythm may be interrupted by gasping, an autoresuscitative behavior characterized by short, intense quasiperiodic decrementing bursts of inspiratory activity (7–9). Gasping is a potential biomarker for survival during cardiac arrest (10, 11). The intense inspiratory efforts can generate large subatmospheric intrapleural pressures that yield large tidal volumes resulting in enhanced coronary perfusion and carotid blood flow to the brain (12–14). Accordingly, we postulate that this interdependence effectively “bootstraps,” or coordinates, peripheral chemoreceptor reflexes, the respiratory rhythm, and enhanced sympathetic outflows.

Carotid chemoreceptors operate through multiple paths in the raphe-pontomedullary respiratory network with its embedded, dynamically configured memory system regulated by serotonin (5-hydroxytryptamine, 5-HT) (4). Enhanced brain stem release of 5-HT (primarily produced in the raphe) follows the onset of severe hypoxia (15, 16). The action(s) of 5-HT on 5-HT_2A/2C_ and 5-HT_4_ receptors and/or substance P on neurokinin-1 receptors maintain inspiratory motor output during resting breathing, and 5-HT can cause the activity of some medullary pre-Bötzinger complex (pre-Böt) neurons to shift to an intrinsic bursting pattern (17–19). Onset of gasp initiation and recovery of eupnea have been shown to be delayed in animal models with disrupted serotonergic signaling (20–23). Notably, acute in vivo triggering of synthetic hM4D_i_ (a modified form of the human M4 muscarinic receptor) inhibitory receptors expressed in serotonergic *Pet1* neurons not only alters baseline cardiorespiratory behaviors but also promotes impaired gasping and reduced survival during apneas in neonatal mice (24).

The brain stem network operations that amplify inspiratory drive during hypoxia and gasping are incompletely understood. Previous studies on neuronal and glial signaling mechanisms for augmented breaths (sighs) and gasping have primarily focused on excitatory inspiratory neurons within the ventral respiratory column (VRC) (9, 25–29). However, the VRC interacts also with other regions including raphe and pontine circuits that modulate breathing through multiple routes (18, 30–35). Thus, our first objective was to test the hypothesis that hypoxia-evoked gasps are amplified through a distributed efference copy mechanism (i.e., corollary discharge as with axon collaterals). This mechanism would be expected to generate coordinated gasp-synchronous discharges in other circuits of the raphe-pontomedullary respiratory network simultaneously.

The chain of VRC inspiratory neurons that generates inspiratory drive is continuously tuned by recurrent excitation and by feedforward and recurrent inhibitory circuits both within and outside of the VRC that actively constrain inspiratory drive (32, 34–39). For example, 5-HT released by the medullary raphe nuclei has been proposed to have a disinhibitory influence on respiratory drive (40, 41). Our second aim was to test the hypothesis that gasping engages a disinhibitory microcircuit within the inspiratory neuron chain to amplify inspiration, thereby enhancing the distribution of gasp drive to multiple targets in the brain and spinal cord. We further propose that these two mechanisms could work in concert to amplify the ballistic, dramatic motor pattern of gasping.

Our approach to produce hypoxia and gasping was ventilation with a gas mixture of 5% O_2_ in a balance of nitrogen (N_2_). Brain stem neuron spike trains were recorded at multiple sites simultaneously during the evoked changes in the respiratory motor pattern and evaluated for firing rate modulations and short-timescale correlations indicative of paucisynaptic functional connectivity.

## METHODS

All experiments were performed according to protocols approved by the University of South Florida’s Institutional Animal Care and Use Committee with strict adherence to all Association for Assessment and Accreditation of Laboratory Animal Care International (AAALAC), National Institutes of Health, National Research Council, and US Department of Agriculture guidelines. The data comprising this work were part of studies on raphe-pontomedullary network organization and chemoreceptor reflex circuits. Complementary results on the connectivity and central and/or peripheral chemoreceptor-evoked responses of a subset of neurons described here have been reported in previous studies (33–36, 42).

### Surgical Protocols

Data were obtained from six adult purpose-bred cats for research (3.0–4.0 kg; 5 females, 1 male; R&R Research, Howard City, MI) initially anesthetized with isoflurane mixed with air (3–5%) or with ketamine hydrochloride (5.5 mg/kg im); all were maintained with 0.5–3.0% isoflurane until decerebration (43). Atropine (0.5 mg/kg im) was administered at the onset of the experiment to reduce mucus secretion in the airways. Animals were artificially ventilated through a tracheal cannula using a mechanical ventilator; all six cats were bilaterally vagotomized to remove vagal afferent feedback from pulmonary stretch receptors and to permit comparisons with prior work. Immediately before decerebration, an anesthetic assessment was performed (33) and animals were neuromuscularly blocked with pancuronium bromide (initial bolus 0.1 mg/kg followed by 0.2 mg/kg/h iv).

Arterial blood pressure, end-tidal CO_2_, and tracheal pressure were monitored continuously. Arterial Po_2_, Pco_2_, and pH were measured periodically, and arterial blood pressure was maintained with solutions of 6% Dextran 70 in 0.9% sodium chloride or 0.04–0.1% dopamine (as needed); sodium bicarbonate solution (8%) was used to correct metabolic acidosis. Diphenhydramine hydrochloride (1.8 mg/kg iv) was administered to reduce mucus secretion in the airways. Dexamethasone (initial bolus of 2.0 mg/kg followed by 4.5 mg/kg/h iv) was dispensed to help prevent hypotension and minimize brain swelling.

### Changes in Chemical Drive: Hypoxia

Transient intervals of hypoxia were produced by ventilation with a gas mixture of 5% O_2_ and 95% N_2_ for 90–905 s; then air was reintroduced into the ventilator. In some cats, during the hypoxic exposure and immediately after, arterial blood pressure support was provided by inflation of an embolectomy catheter placed in the descending aorta. For all but one cat, supplemental 100% O_2_ was added to the ventilation gas after blood pressure support was withdrawn.

### Data Acquisition

Efferent phrenic nerve discharge was used to identify the phases of breathing and as an indicator of respiratory drive and stimulus effectiveness. Extracellular neuronal activity was acquired from three multielectrode arrays with individually adjustable (submicrometer steps) high-impedance tungsten microelectrodes (1-μm tip diameter; 10–12 MΩ, 72 total electrodes). Electrode placement was guided by anatomical landmarks (obex, brain stem midline), appropriate stereotaxic coordinates derived from Berman (44), and the results of previous studies from our laboratory (see Fig. 1 in Ref. 45, Refs. 33, 34, 36, 38) and others (46, 47). Neurons in the vicinity of the medullary raphe nuclei, the ventral respiratory column, and the pontine respiratory group were recorded. Spike trains from unique individual neurons were isolated offline with an interactive spike sorting software package (48), and coordinates of electrode tips (i.e., recording sites) were mapped into the three-dimensional (3-D) space of a computer-based brain stem atlas derived from Berman (44).

### Data Analysis

Standard firing rate histograms and phase-normalized respiratory cycle-triggered histograms (CTHs) were calculated for each spike train using activity recorded during a 30-min normoxic control period in air; neurons were classified as respiratory modulated if either of two complementary statistical tests rejected the null hypothesis (*P* < 0.05) as previously described (49–51). Respiratory-modulated neurons were classified as inspiratory (I) or expiratory (E) according to the part of the cycle during which the cell was most active (52); non-respiratory-modulated (NRM) cells had no preferred phase of maximum activity. Cells were labeled phasic if their firing probability was essentially zero during any part of the control respiratory cycles, or tonic otherwise. All CTHs were individually scaled to their maximum firing rate.

Functional connectivity within and among the brain stem regions studied was detected and evaluated with cross-correlation histograms (35, 42) calculated for all pairs of simultaneously monitored neurons. The cross-correlation histogram gives an estimate of the probability that an action potential in one (reference) spike train will be preceded or followed by action potentials in a second (target) spike train. Significant peak or trough features in cross-correlograms were identified with Monte Carlo tests using surrogate spike trains (53) with gamma-distributed interspike intervals; the false discovery rate was kept < 0.05. The shape parameter of the gamma distribution was estimated from the data (54). A detectability index, calculated as the maximum amplitude of feature departure from background activity divided by the standard deviation of the correlogram noise, that was >3.0 indicated a significant correlogram feature (55, 56). All offset-feature data are presented with a positive time lag. Correlation feature maps, including directed graphs representing inferred connectivity among simultaneously monitored neurons, were generated (34).

## RESULTS

The results describe neuronal firing rate dynamics and correlational signatures of functional connectivity during the various respiratory motor patterns induced by transient ventilation with a hypoxic gas mixture (5% O_2_, balance N_2_). For this investigation, a total of 467 neurons (268 VRC, 90 raphe, and 109 pontine cells) were isolated and evaluated in the six vagotomized cats studied. Recording sites in the pons were located from the caudal border of the inferior colliculus to 1.0 mm posterior to it, 3.7 to 6.1 mm lateral to the midline, and 1.1 to 5.6 mm below the dorsal surface of the pons. Recording sites in the raphe were located 2.2 to 5.6 mm rostral to the obex, within 0.2 mm on either side of the midline, and 1.0 to 5.0 mm below the dorsal surface. Recording sites in the VRC were located 1.4 mm caudal to 6.9 mm rostral to the obex, 3.0 to 4.7 mm lateral to the midline, and 1.9 to 4.8 mm below the surface of the medulla. Pairs of neurons (*n* = 20,521) were assessed for evidence of short-timescale functional interaction, with 46% of neurons exhibiting functional interactions with at least one other neuron (Table 1). The characteristics and incidence of hypoxia-evoked changes in respiratory motor patterns as indicated by altered phrenic nerve activity are summarized in Table 1.

**Table 1.** Order of hypoxia-induced changes in motor patterns as indicated by altered phrenic nerve activity

Animal	Hypoxia Duration(5% O_2_, 95% N_2_)	Motor Pattern Sequence				No. of Neurons Functionally Connected with at Least 1 Other Neuron	No. of Neurons Recorded	Recorded Neurons with at Least 1 Functional Interaction
		Augmentation	Depression	Apnea	Autoresuscitative efforts			
*1*	268 s, 305 s (2 trials)	1st	2nd	3rd	4th	48	116	41%
*2*	488 s	1st	2nd			41	110	37%
*3*	277 s	1st	2nd	3rd	4th	22	54	41%
*4*	905 s	2nd	1st	3rd	4th	25	53	47%
*5*	480 s	1st	2nd	3rd		21	42	50%
*6*	90 s	1st	2nd	3rd	4th*	60	92	65%
Total						217	467	46%

Not all motor patterns were expressed in every animal. Functional interactions were indicated by significant features in cross-correlations of spike train pairs; features with a detectability index ≥ 3 were considered to be significant (55, 56). For example, in *animal 1*, 48 of 116 recorded neurons were functionally connected with at least 1 other recorded neuron. *Animal died before recovery from hypoxia.

### Sequence of Motor Patterns during Ventilation with a Hypoxic Gas Mixture

**Figure 1.**
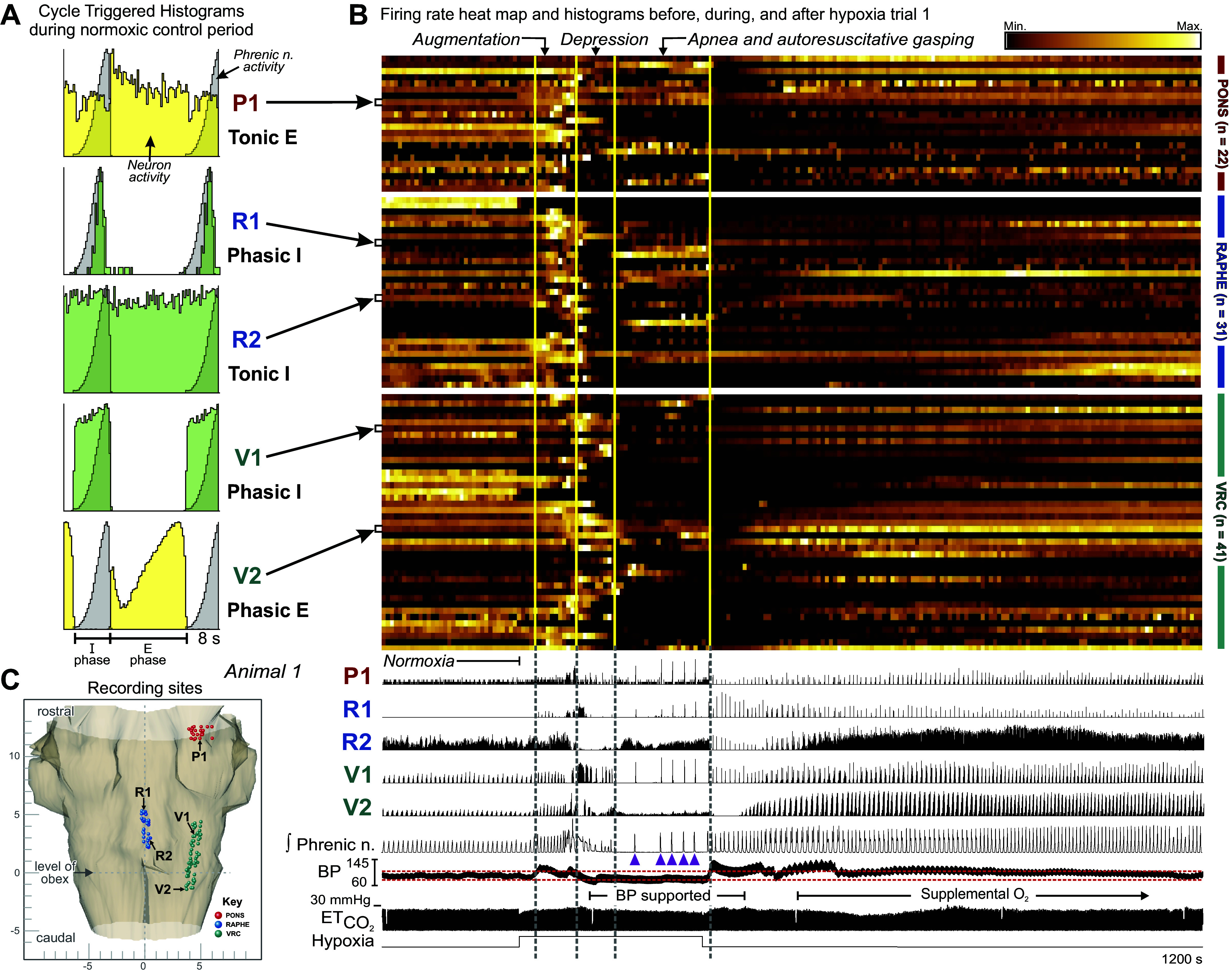
Neurons change their firing rates and patterns of activity during a hypoxia-evoked sequence of motor patterns produced by ventilation with 5% O_2_. *A*: selected phase-normalized respiratory cycle-triggered histograms (CTHs) show the firing patterns of 1 pontine cell (*P1*), 2 ventral respiratory column (VRC) neurons (*V1*, *V2*), and 1 midline raphe cell (*R2*) during the normoxic control period. *Cell R1* was not active during control; data for its CTH were collected during and after the hypoxic exposure. Inspiratory (I) and expiratory (E) phase durations are indicated beneath the CTHs. *B*: firing rate heat map (*top*, with luminance proportional to respective normalized rates) and firing rate histograms for 94 of 116 neurons recorded in *animal 1* during *hypoxia trial 1*, together with integrated phrenic nerve activity, blood pressure (BP), and end-tidal CO_2_ (${\text{ET}}_{{\text{CO}}_{2}}$) (*bottom*); hypoxic gas ventilation was applied for 268 s. Gasp bursts are indicated by purple arrowheads. Brain stem regions are indicated on *right* of the traces. Blood pressure was supported by the inflation of an embolectomy balloon catheter in the descending aorta as marked; red dashed lines in blood pressure trace indicate blood pressure range seen during preceding control interval. Note that 100% O_2_ was added to the ventilation gas after blood pressure support was withdrawn. These data document a variety of changes in neuronal firing rate and pattern as the drive to breathe is first augmented and then depressed to an apneic state interrupted by a gasping motor pattern; diverse rates occurred during recovery from hypoxia as well. Vertical lines through the heat map and rate histograms separate periods of respiratory augmentation, depression, and apnea and autoresuscitative gasping motor patterns. *C*: dorsal view of the stereotaxic coordinates of electrode tips (i.e., recording sites) in *animal 1* monitored simultaneously in the VRC (green), pons (red), and midline raphe (blue) mapped into the brain stem atlas.

**Figure 2.**
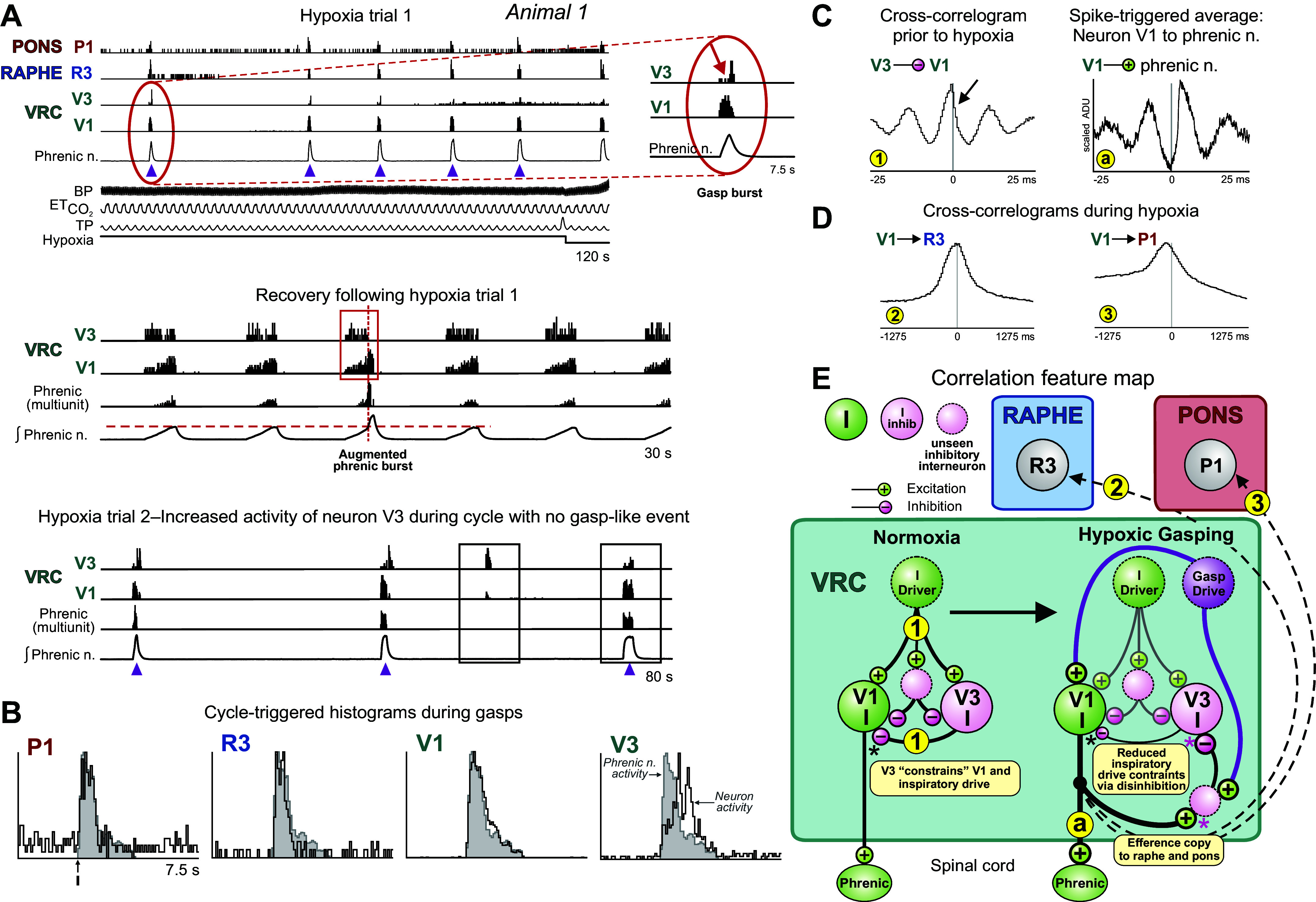
Firing dynamics and synchronization of neuron bursting during hypoxia-evoked gasping inform a model of gasp drive amplification. *A, top*: firing rate histograms of selected neurons recorded in *animal 1* detail transient bursts in the 3 monitored regions of the brain stem respiratory network during gasp discharges seen in phrenic motor output (purple arrowheads) consistent with distributed efference copies of inspiratory drive to pontine and medullary raphe neurons. *Inset*, detail of reduced activity in *neuron V3* (red arrow) during peak burst rate in *cell V1*. BP, blood pressure; ${\text{ET}}_{{\text{CO}}_{2}}$, end-tidal CO_2_; TP, tracheal pressure; VRC, ventral respiratory column. *Middle*: firing rate histograms during recovery from *hypoxia trial 1* highlighted by a red box show absence of spiking in *neuron V3* and increased firing in *neuron V1* during a period of augmented phrenic burst activity; horizontal dashed red line indicates peak integrated phrenic activity without augmentation. Also shown is a measure of multiunit phrenic activity derived from the integrated phrenic nerve signal. *Bottom*: firing rates during gasp bursts occurring in *hypoxia trial 2* show reciprocal rate changes in the 2 neurons during a “missed” phrenic event (*left* box) and during a subsequent apneustic burst (*right* box). *B*: cycle-triggered histograms (CTHs) show an augmenting pattern for *neuron V3* and decrementing patterns for *neurons V1*, *P1*, and *R3* during gasping. *C, left*: cross-correlogram for *neurons V3* and *V1* includes an asymmetrical central peak flanked by trough-peak features; data collected before hypoxic trials. The detectability index value for the central peak is 19.4. Number of spikes for each neuron used to calculate cross-correlograms: *V1*: 102,124; *V3*: 30,059. *Right*: spike-triggered average of full wave-rectified left (contralateral) phrenic nerve activity triggered by VRC *cell V1*. ADU, analog-to-digital units. *D*: cross-correlograms constructed from activity during and after the hypoxia trials are consistent with efference copies of inspiratory drive. Detectability index values for the peaks in each histogram: *2*: 10.95; *3*: 3.27. Number of spikes for each neuron: *P1*: 7,313; *V1*: 102,124; *R3*: 1,259. *E*: correlation feature map and other interactions suggested by the results and prior work; see text. Numbers and letters in small yellow circles denote cell pair correlograms and spike-triggered averages, respectively.

The sequential generation of motor patterns typically observed in response to ventilation with the hypoxic gas mixture included periods of hypoxia-evoked augmentation, depression (apneusis and sighs), and apnea and gasping, albeit not all motor patterns were noted in all experiments (Table 1). Figure 1 and Fig. 2 present the data obtained from *animal 1* before, during, and after the hypoxic exposure in *hypoxia trial 1*. Neuronal spike trains were monitored concurrently at multiple sites in the VRC, raphe, and pons; the location of each neuron’s recording site (i.e., 3-D coordinates of the recording electrode tip) was mapped into a brain stem atlas (Fig. 1*C*). A heat map of neuronal firing rates measured simultaneously in 94 neurons before, during, and after hypoxia highlights the variety of rate modulations during the response to and recovery from hypoxic ventilation (Fig. 1*B*, *top*); dark tones indicate minimal firing rates and brighter tones indicate faster firing rates. Firing rate histograms for a subset of the neurons during the same time period, together with integrated phrenic nerve activity, blood pressure, and end-tidal CO_2_, document changes in the drive to breathe. Each of the three brain stem regions included neurons with firing rates tightly coupled to the gasp motor burst pattern evident in the phrenic record; note that bursts from *neurons P1*, *V1*, and *R1* were synchronous with the bursts in phrenic discharge (Fig. 1*B*, *bottom*, purple arrowheads). Respiratory CTHs calculated during the normoxic control period show that neurons with either inspiratory or expiratory discharge patterns exhibited this gasp-associated modulation of firing rate (Fig. 1*A*).

Across all animals, 40% (10 of 25) of neurons with bursts during gasps were not inspiratory cells during the normoxic control period; six neurons were expiratory cells, one was most active at the inspiration-to-expiration transition, and three were not breathing modulated. In general, during hypoxic exposure we observed a period of augmented breathing characterized by increased phrenic amplitudes and/or frequency, followed by a depression that could include apneusis, then finally apnea and gasping; vertical lines though the firing rate heat map and histograms separate these changes in motor patterns (Fig. 1*B*, *top*). Not all animals exhibited each phase, and the phases were not always in this order; Table 1 details the presence and order of periods of the augmentation, depression, apnea, and gasping motor patterns for each animal during hypoxic ventilation.

Higher-temporal resolution firing rate histograms for the same animal show gasp-related bursts indicative of widespread engagement of the respiratory network during and immediately following the hypoxic exposure trial. Gasp-patterned bursts in phrenic motor output were associated with distinct and diverse firing rate profiles in VRC inspiratory neurons. For example, high firing rates in *neuron V1* were temporally congruent with transient declines in firing probability in *neuron V3* (Fig. 2*A*, *inset*). Figure 2*A*, *middle*, shows an augmented phrenic burst (above the red dashed line) at the end of the inspiratory activity coincident with increased *neuron V1* activity, yet *neuron V3* was quiet during the augmented phrenic burst (red box). Conversely, at times when *neuron V1* has decreased firing (Fig. 2*A*, *bottom*, left box), *neuron V3* continues to burst without diminution. During the apneustic burst in the right box, however, the neuron firing activities reverse, and *neuron V1* increases firing while the firing rate of *neuron V3* declines. The cross-correlogram constructed from activity before hypoxia for this pair of neurons had a broad asymmetrical central peak (Fig. 2*C*, *left*, arrow) and adjacent bilateral troughs and peaks; the average of the phrenic nerve signal triggered by spikes in *neuron V1* also displayed bilateral peaks and troughs (Fig. 2*C*, *right*), possibly due to coordinated oscillations. These results are consistent with contemporary models of the VRC inspiratory neuron chain and suggest that *neurons V1* and *V3* received excitation and delayed inhibition via upstream I-driver neurons under normoxic conditions (Fig. 2*E*, *left*).

During the interval that included hypoxia-evoked gasping, synchronously discharging pontine and raphe *neurons P1* and *R3* had decrementing inspiratory profiles similar to VRC *neuron V1* (Fig. 2*B*); *neuron V3* had an augmenting inspiratory pattern. Cross-correlograms calculated from these data for trigger *neuron V1* and targets *R3* and *P1* had broad central peaks (Fig. 2*D*, *correlograms 2* and *3*), consistent with efferent copies of an enhanced inspiratory drive produced by disinhibition and transmitted to the raphe and pons (Fig. 2*E*, *right*, and *correlograms 2* and *3*).

### Functional Connectivity of Neurons with Hypoxia-Evoked Augmentation, Apneusis and Sighs, and Gasplike Motor Patterns

**Figure 3.**
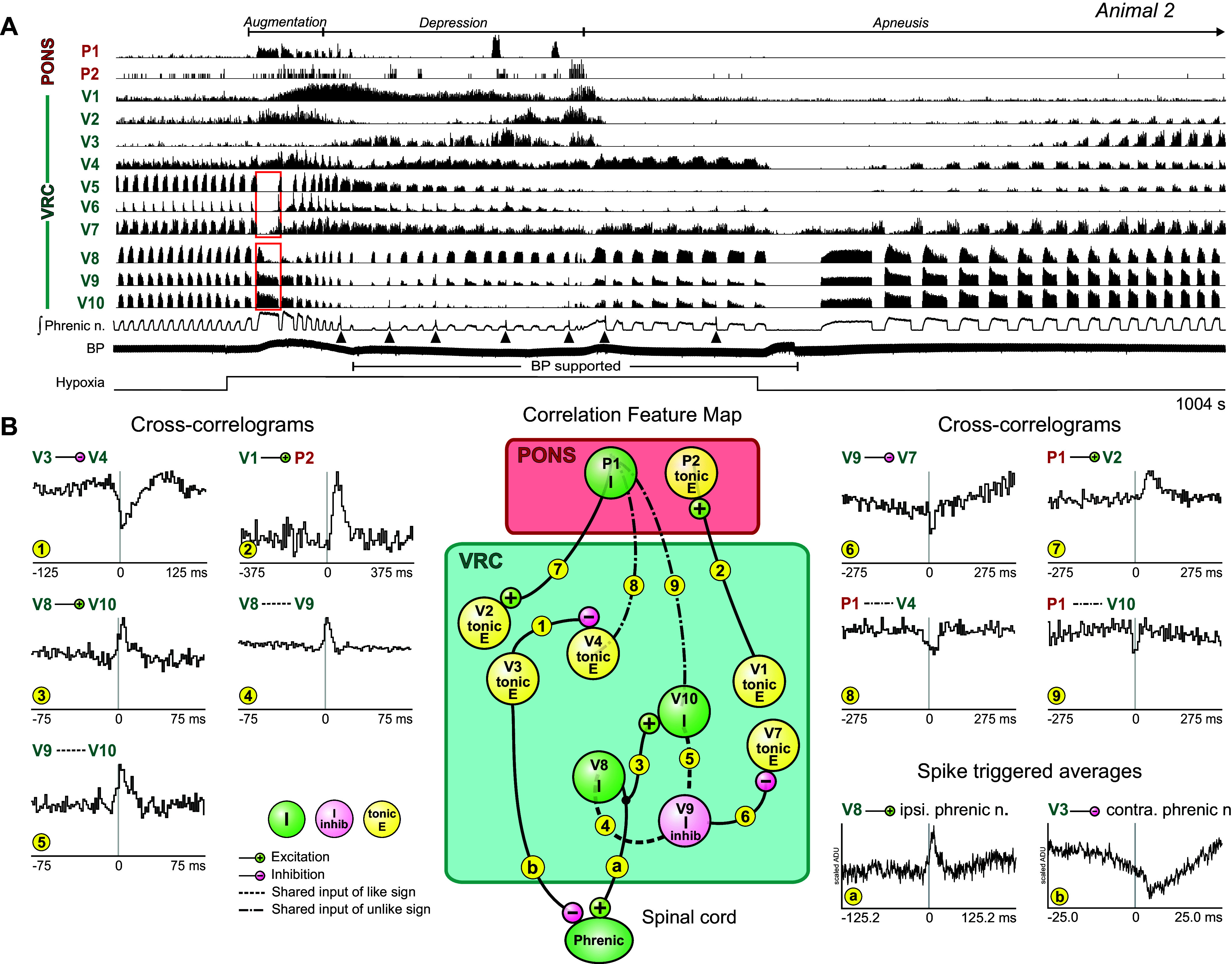
Hypoxia evokes an apneustic motor pattern with augmented bursts. *A*: firing rate histograms of pontine and bilateral ventral respiratory column (VRC) neurons together with integrated phrenic nerve and arterial blood pressure (BP) before, during, and after ventilation with 5% O_2_ recorded in *animal 2*. Apneustic patterns and augmented bursts (black arrowheads) were detected. Red boxes point out changes in firing patterns of some VRC neurons during an apneustic breath. Bar beneath blood pressure trace denotes period of blood pressure support with inflation of an embolectomy balloon catheter in the descending aorta. *B*, *left* and *right*: cross-correlograms for indicated pairs of neurons. Detectability index values for troughs or peaks in each histogram: *1*: 8.78; *2*: 6.85; *3*: 6.29; *4*: 13.09; *5*: 6.49; *6*: 3.51; *7:* 5.32; *8*: 5.49; *9*: 3.59. Number of spikes for each neuron: *P1*: 9,824; *P2*: 384; *V1*: 51,534; *V2*: 17,534; *V8*: 201,037; *V7*: 109,890; *V4*: 52,680; *V3*: 133,080; *V10*: 68,479; *V9*: 198,262. Spike-triggered averages of full wave-rectified ipsi- and contralateral phrenic nerve activity show offset peak and trough features for trigger *neurons V8* and *V3*, respectively. ADU, analog-to-digital units. *Center*: correlation feature map summarizing simple interpretations of cross-correlogram features; see text for details. Numbers and letters in small yellow circles denote cell pair correlograms and spike-triggered averages, respectively.

In some experiments, ventilation with the hypoxic gas mixture evoked an apneustic motor pattern with intermittent augmented bursts, but no gasping. Figure 3 presents data obtained from *animal 2* to illustrate this hypoxia-evoked motor pattern and the associated changes in neuronal activities. During the initial period of enhanced inspiratory drive, simultaneously monitored pontine and VRC neurons had firing patterns with active intervals or pauses that matched the durations of concurrent apneustic inspiratory bursts (Fig. 3*A*). The firing pattern of I *neuron V8* changed from augmenting during the respiratory cycles immediately before the hypoxic exposure to decrementing during the apneustic cycles; although the neuron fired throughout apneustic inspiration, its highest rate occurred during the first half of the I phase (Fig. 3*A*, bottom red box). In contrast, the firing rates of two decrementing inspiratory neurons, *V9* and *V10*, were more constant throughout the apneustic I phase, and they maintained their overall decrementing pattern of activity (Fig. 3*A*, bottom red box). Concurrently, augmenting expiratory *neuron V5*, postinspiratory (or decrementing expiratory neuron) *V6*, and tonic expiratory *neuron V7* exhibited reduced firing probability throughout the apneustic inspiratory phase (Fig. 3*A*, top red box).

As the hypoxic episode continued, phrenic activity was depressed and augmented bursts (sighs) located near the end of the inspiratory activity occurred intermittently (Fig. 3*A*, black arrowheads). The average of the rectified phrenic nerve signal triggered by I *neuron V8* had an offset peak with a positive lag, a result consistent with a premotor function (Fig. 3*B*, *bottom right*, *spike-triggered average a*). In contrast, the average of the rectified phrenic nerve signal triggered by tonic E *neuron V3* had an offset trough suggestive of functional inhibition of inspiratory drive (Fig. 3*B*, *bottom right*, *spike-triggered average b*). The firing rates of tonic E *neurons V3* and *V4* fluctuated as the hypoxic exposure continued, with the greatest firing rates in each tending to occur at different times. Notably, an offset cross-correlogram trough supported an inhibitory action of *neuron V3* upon *neuron V4* (Fig. 3*B*, *histogram 1*).

Cross-correlation analysis of other neuron pairs revealed features suggestive of various VRC and pontine network operations and supported observed firing rate changes. The functional interactions inferred from correlogram features are summarized in the corresponding correlation feature map (Fig. 3*B*, *center*). VRC tonic E *neuron V1* triggered a cross-correlogram with pontine tonic E *neuron P2* featuring an offset peak (Fig. 3*B*, *histogram 2*). The offset peak in the correlogram for pair *V8-V10* (Fig. 3*B*, *histogram 3*) and asymmetric central peak features for pairs *V8-V9* and *V9-V10* (Fig. 3*B*, *histograms 4* and *5*) are consistent with interactions between, or shared influences among, this trio of neurons. We note that *neurons V10* and *V9* had decrementing discharge patterns during preceding control inspiratory bursts and that the cross-correlogram triggered by *V9* for target tonic E *neuron V7* had an offset trough feature consistent with a functional inhibition of the tonic E neuron that likely continued to operate during the apneustic bursts (Fig. 3*B*, *histogram 6*) as shown in Fig. 3*A*. Cross-correlogram features for pontine trigger *neuron P1*, also active during apneustic bursts, included an offset peak with target tonic E *neuron V2* (Fig. 3*B*, *histogram 7*) and central troughs with targets *V4* and *V10* (Fig. 3*B*, *histograms 8* and *9*). These latter features suggest unobserved shared influences having opposite actions upon the elements of each pair.

### Differential Modulation of Neuron Firing Rates during Augmentation, Hypoxic Gasping, and Gasp-Associated Step Increments in Blood Pressure

**Figure 4.**
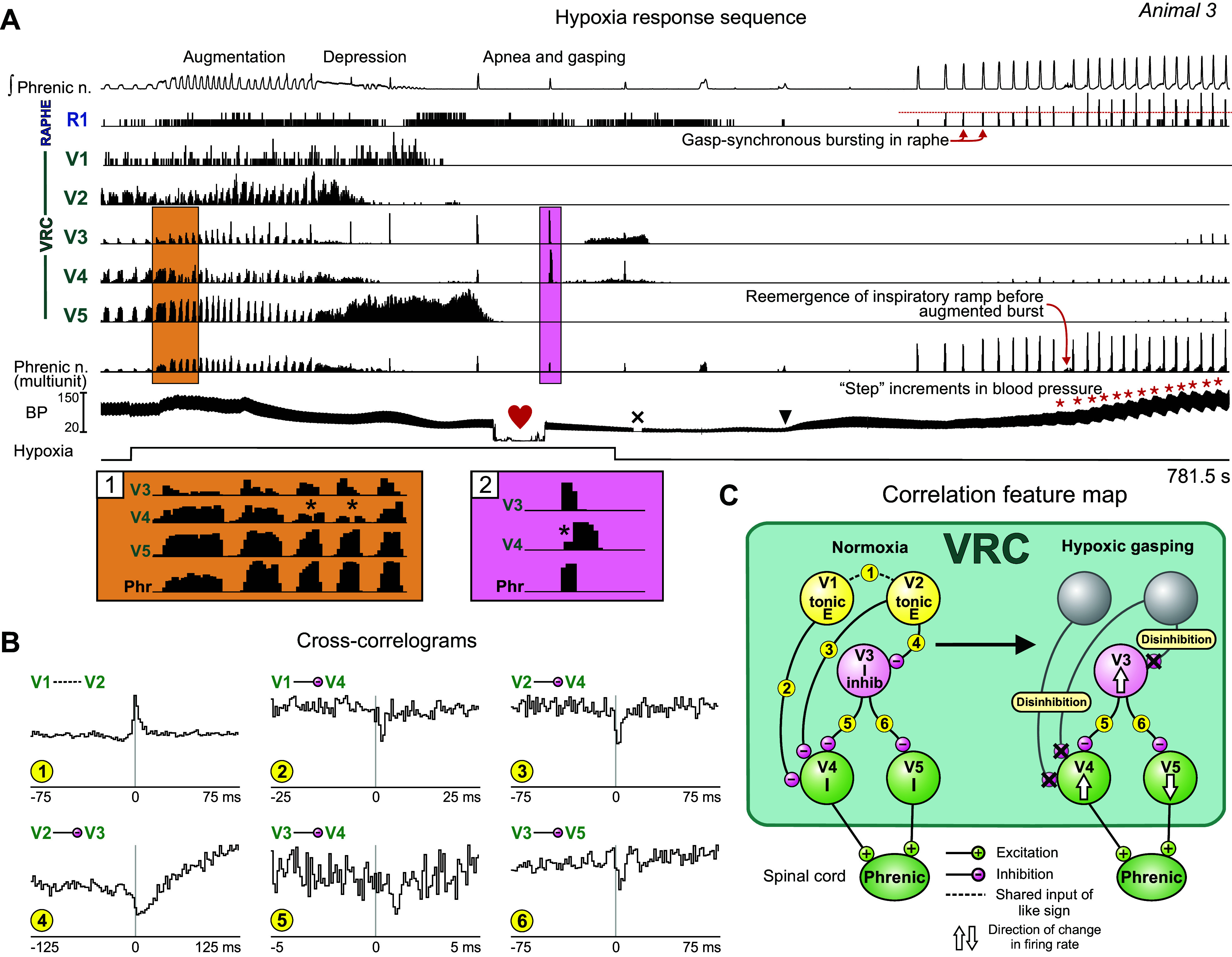
Stepwise increments in blood pressure are synchronous with gasps evoked by hypoxia. *A*: integrated phrenic activity, neuronal and multiunit phrenic firing rate histograms, and blood pressure (BP) before, during, and after hypoxic gas ventilation. Firing rate histograms show changes in discharge patterns of ventral respiratory column (VRC) neurons during hypoxia. *Insets 1* and *2*, details of firing dynamics highlighted in *A*. Note stepwise increases in systemic arterial blood pressure associated with bursts in VRC and raphe neurons (red asterisks). Black arrowhead denotes time of 10-mL dextran bolus; “×” indicates artifact removal from the BP trace. Heart symbol indicates time of blood collection for blood gas measurement (the trace is flat because blood was diverted from the pressure transducer during collection): Po_2_ = 31.8 mmHg; pH =7.41; Pco_2_ = 25.6 mmHg. *B*: cross-correlograms for the indicated pairs of neurons. Detectability index values for troughs or peaks in each histogram: *1*: 25.01; *2*: 7.81; *3*: 6.64; *4*: 7.41; *5*: 6.09; *6*: 4.92. Number of spikes for each neuron: *V3*: 53,680; *V1*: 59,942; *V4*: 113,205; *V5*: 175,482; *V2*: 63,600. *C*: correlation feature map summarizes inferred relationships and indicates mechanisms of interaction and changes in cell firing rate associated with the amplification of inspiratory drive as described in the text. Numbers in small yellow circles denote cell pair correlograms. E, expiratory; I, inspiratory.

Ventilation with hypoxia in *animal 3* evoked a sequence of motor patterns that began with augmented inspiratory drive followed by apneusis and respiratory depression, leading to apnea and gasping (Fig. 4*A*). The VRC neurons monitored during this motor pattern sequence included decrementing and augmenting inspiratory and expiratory neurons. In addition to an increased respiratory cycle frequency, differential changes in the firing rates of some I neurons were apparent during the augmented drive (Fig. 4*A*, orange box). The peak rate of *neuron V5* increased, whereas that of *neuron V4* declined, in some cycles to such an extent that only two small bursts were apparent at the start and end of the inspiratory phase (Fig. 4*A*, *inset 1*, asterisks). Concurrent with these altered patterns, the peak rate of E *neuron V2* declined as that of I *neuron V3* increased.

A central peak feature in the cross-correlogram for tonic E *neurons V1* and *V2* suggests a shared source of drive (Fig. 4*B*, *correlogram 1*), yet neither neuron was active during the period of apnea and subsequent gasps. The cross-correlograms triggered by *neurons V1* and *V2* with target inspiratory *neurons V4* and *V3* were characterized by offset troughs, implying convergent as well as divergent functional inhibitory influences (Fig. 4*B*, *correlograms 2–4*). Cross-correlograms triggered by *neuron V3* had offset trough features consistent with divergent functional inhibition of *neurons V4* and *V5* (Fig. 4*B*, *correlograms 5* and *6*); the neuronal activities presented in *insets 1* and *2* (asterisks) of Fig. 4*A* show cycles in which a reduction in the firing rate of *neuron V4* coincides with the peak rate of *neuron V3*. Collectively, these data suggest that loss of activity in *neurons V1* and *V2* during apnea resulted in reduced inhibition of *neurons V3* and *V4* and led to differential tuning of the firing rates of *neurons V4* and *V5*, both of which triggered phrenic signal averages featuring offset peaks suggestive of premotor functions (not shown). These correlation features and inferred changes to the circuit during hypoxic gasping are summarized in Fig. 4*C*. With the return of air ventilation and support of blood pressure (black arrowhead on BP trace in Fig. 4*A* denotes 10-mL dextran injection), gasplike bursts emerged in the phrenic nerve, accompanied by steplike increases in arterial blood pressure (Fig. 4*A*, red asterisks). Concurrent bursts occurred in raphe *neuron R1* and VRC neurons *V3*, *V4*, and *V5*; increased firing rates in these neurons were associated with the increases in blood pressure.

**Figure 5.**
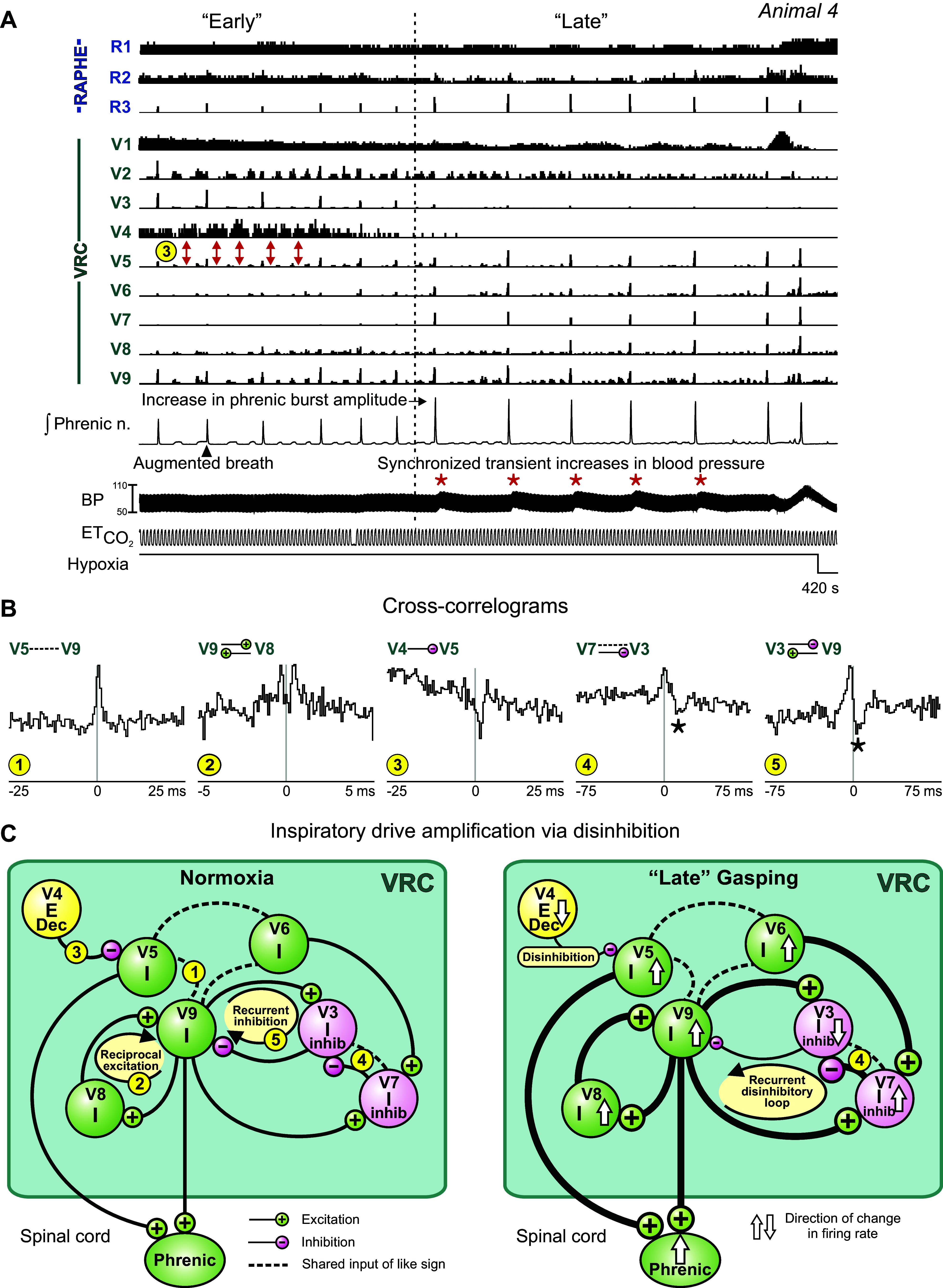
Changes in phrenic burst amplitude correspond with “early” and “late” gasp-synchronous bursting neuron groups during hypoxia. *A*: firing rate histograms showing detail of firing rates of raphe and ventral respiratory column (VRC) neurons during hypoxia-evoked gasp bursts. Note the transition from lower-amplitude to higher-amplitude bursts of integrated phrenic activity and corresponding changes in burst amplitudes of “early” and “late” responding VRC and raphe neurons. Synchronized transient increases in systemic arterial blood pressure (BP) (red asterisks) were associated with each of the “late” gasp bursts. Red double-headed arrows indicate asynchronous firing patterns of *cells V4* and *V5*; functional inhibition of *V5* by *V4* is inferred by the positive-lag trough feature in *cross-correlogram 3* in *B*. ${\text{ET}}_{{\text{CO}}_{2}}$, end-tidal CO_2_. *B*: cross-correlograms for the indicated pairs of neurons. Detectability index values for troughs or peaks in each histogram: *1*: 5.8; *2*: 5.00; *3*: 5.77; *4*: 6.71; *5*: 9.42. Number of spikes for each neuron used to calculate cross-correlograms: *V3*: 81,986; *V4*: 43,094; *V5*: 322,707; *V7*: 86,003; *V8*: 103,220; *V9*: 170,819. *C*: correlation feature map summarizes inferred relationships and indicates mechanisms of interaction and changes in cell firing rate associated with the amplification of inspiratory drive as described in the text. Numbers in small yellow circles denote cell pair correlograms. E, expiratory; I, inspiratory.

A similar sequence of respiratory depression and apnea leading to gasping, with the gasp bursts coordinated with increments in blood pressure, was observed in *animal 4* (Fig. 5). Ventilation with 5% O_2_ evoked an initial apnea (not shown) followed by gasping discharges intermingled with augmented breath sequences; peak firing rates declined with successive bursts in some neurons and increased in others (Fig. 5*A*; e.g., VRC *neurons V3* and *V6*, respectively). Several of the larger gasp-patterned phrenic bursts followed a series of smaller, yet successively incrementing, inspiratory phase discharges (e.g., VRC *neuron V9*). During the latter part of the hypoxic exposure period, transient increases in blood pressure (red asterisks) were associated with increased gasp-burst amplitude in the phrenic nerve and gasp-synchronous firing in raphe I *neuron R3* and VRC I *neurons V5*, *V6*, *V7*, *V8*, and *V9*. Slow oscillations in the firing rates of other raphe (*R1* and *R2*) and VRC (e.g., *V1* and *V2*) neurons synchronized with the fluctuations in blood pressure were also detected during this “late” response period. Some neurons exhibited a greatly reduced firing rate during the late response (*neuron V3*) or stopped firing altogether (*neuron V4*).

The cross-correlogram for augmenting inspiratory *neurons V5* and *V9* had a central peak feature consistent with shared excitation (Fig. 5*B*, *correlogram 1*); offset peaks in their spike-triggered averages of the rectified phrenic nerve signal suggest a premotor function for the two neurons (not shown). The dual peaks in the correlogram for neuron pair *V9-V8* (Fig. 5*B*, *correlogram 2*) are consistent with a reciprocal excitatory relationship, one operation appropriate for a role in inspiratory drive amplification (Fig. 5*C*). Correlational signatures of functional connectivity among the monitored neurons suggest that inspiratory chain-mediated disinhibition contributes to gasp drive amplification. For example, the offset trough feature in the cross-correlogram triggered by *neuron V4* for target *neuron V5* (and the inferred functional inhibition of *V5* by *V4*; Fig. 5*B*, *correlogram 3*) is consistent with the activities of these two neurons during the early period of response, when the firing patterns of the two neurons are asynchronous (Fig. 5*A*, *correlogram 3*, red double-headed arrows), as well as during the late period, when the firing rate of inhibitory *neuron V4* declines precipitously and the gasp-synchronous firing of *neuron V5* becomes greater, indicating amplification of inspiratory drive via disinhibition (Fig. 5*C*). In this dataset, we identified 24 inhibitory relationships between neurons. Of these, eight are examples of disinhibition of activity during gasps, i.e., the absence of activity in an inhibiting cell corresponded to gasping activity in the target cell. Seven of the eight neuron pairs included an expiratory VRC cell and a target inspiratory VRC cell.

Coordinated inspiratory *neurons V6* and *V9* each triggered a correlogram with target *neuron V7* that featured an offset peak (not shown), a result consistent with convergent excitation. *Neuron V7* was identified as an element of a correlational inspiratory neuron chain; it triggered a correlogram with *neuron V3* featuring a positive-lag offset trough relative to a central peak (Fig. 5*B*, *correlogram 4*, asterisk). A similar feature set for pair *V3-V9* (Fig. 5*B*, *correlogram 5*) suggests a process of recurrent disinhibition to further amplify the inspiratory drive generated by *neuron V9*. Inspection of the firing rate histograms of these inspiratory neurons revealed changes consistent with simple interpretations of the short-timescale correlations: as the firing rates of *neurons V6* and *V7* increased during the late response interval, that of *neuron V3* was reduced. The correlation feature map (Fig. 5*C*) summarizes the inferred relationships among these neurons as well as the mechanisms of interaction and directions of change in firing rate associated with the amplification of inspiratory drive as described above.

## DISCUSSION

The results show that acute hypoxia evokes the sequential generation of motor patterns for increases in amplitude and frequency, apneusis and augmented bursts (sighs), apnea, and gasping, with corresponding changes in raphe-pontomedullary circuits of the respiratory network. In a simple model, gasp drive would be generated primarily by excitatory inspiratory VRC neurons rather than through a coordinated reconfiguration of a distributed brain stem network with diverse and changing neuronal patterns. However, the present data are consistent with a more complex model. Correlational signatures of neuronal interactions and altered firing rates support the hypothesis that gasp drive is amplified by a disinhibitory inspiratory chain microcircuit; this drive is distributed via efference copy (e.g., axon collaterals) to generate concurrent bursts in raphe and pontine neurons, which may be coordinated with steplike increments in arterial blood pressure.

### Initial Augmentation

Carotid chemoreceptors contribute to the generation of the initial augmentation with hypoxia, expressed as increases in integrated phrenic nerve amplitude and in respiratory cycle frequency (4). In carotid-deafferented cats, augmentation generated by central mechanisms is characterized by high frequency and low tidal volumes in an O_2_ concentration-dependent manner (57). In the present study, initial augmentation was associated with diverse modulations of firing rate in putative premotor inspiratory neurons. Identified functional connectivity suggested that this motor pattern was shaped by tuning of inhibition by expiratory and inspiratory neurons (Fig. 4). The involvement of raphe circuits in the generation of respiratory augmentation has been suggested by prior work (58–60); we observed numerous interactions between raphe and VRC neurons.

### Apneusis

Persistent hypoxia disrupts neuronal metabolism, leading to a general decline in neuronal activity and the transition to prolonged apneustic inspiratory bursts, some ending with fictive augmented breaths or sighs due to cooperative neuronal and glial signaling (9). The apneusis, indicative of delayed inspiratory-to-expiratory phase switching, may be a consequence of diminished glycinergic inhibition of inspiratory neurons by postinspiratory neurons (61); 5-HT_1A_ receptor agonists can switch this “disturbance” of the respiratory rhythm back to a normal control pattern (62, 63).

### Amplification and Distribution of Gasp Drive

**Figure 6.**
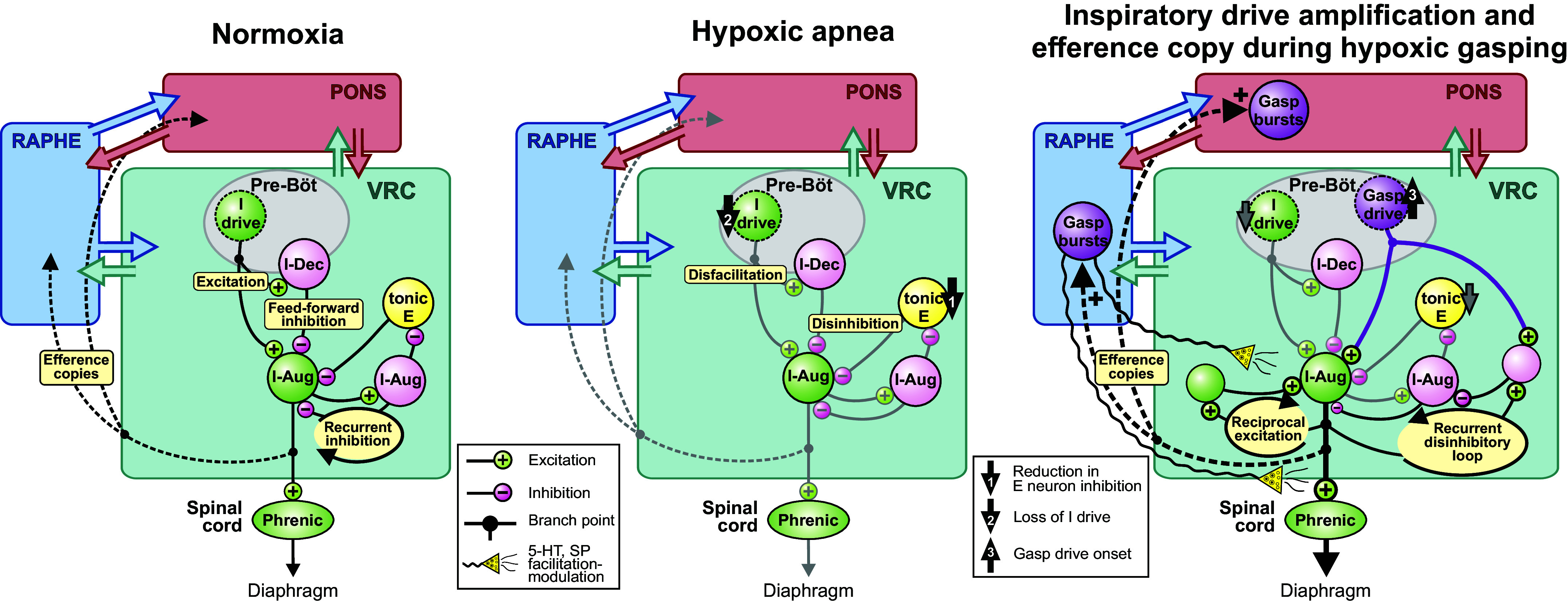
Graphical summary of hypotheses and circuit operations suggested by the results. *Left:* simplified model of functional correlations and circuit mechanisms during normoxia. E, expiratory; I, inspiratory; pre-Böt, pre-Bötzinger complex; VRC, ventral respiratory column. *Center:* a reduction in expiratory neuron inhibition and a loss of inspiratory drive (*arrows 1* and *2*), respectively, result in augmentation and depression of inspiratory drive, leading to hypoxic apnea. *Right:* the onset of gasp-driver neuron bursting (*arrow 3*) excites downstream inspiratory premotor neurons. Efference copies (i.e., corollary discharge such as that which occurs with axon collaterals) of the inspiratory drive engage raphe and pontine circuits in a distributed network of coordinated gasp-related bursting (*right*; dashed lines) in an attempt to generate autoresuscitative efforts. See text for further discussion. I-Dec (I-Aug), peak neuronal firing rate occurred during the first (second) half of the inspiratory phase; SP, substance P.

Prior experiments and computational models, particularly work on persistent sodium currents, have documented a functional simplification of the VRC network during the transition to gasp generation (25, 64–68). This is supported by a recent report by Bush and Ramirez (69), in which many VRC neurons were simultaneously recorded and neuronal population dynamics were represented in a multidimensional space. Stereotyped VRC “rotational” dynamics were observed during eupnea; these were continuously reconfigured during severe hypoxia, ultimately collapsing into all-or-none ballistic trajectories during gasping, suggesting a dissolution of the normal VRC dynamics as hypoxia progresses to gasping (69). Such an “all hands on deck” process for autoresuscitative gasping would involve several mechanisms reflecting the coordinated effort of a reconfigured distributed network, including neurons that change discharge pattern during hypoxic gasping (e.g., *neuron P1* in Fig. 1 and Fig. 2). The functional connectivity, altered firing rates, efference copy of gasp drive, and coordinated step increments in blood pressure we report here support a distributed brain stem network model for amplification and broadcasting of inspiratory drive during autoresuscitative gasping that begins with a reduction in expiratory neuron inhibition followed by a loss of inspiratory drive during hypoxic apnea (Fig. 6, *center*, *arrows 1* and *2*).

The onset of gasp-driver neuron bursting (Fig. 6, *right*, *arrow 3*) excites downstream inspiratory premotor neurons. Although the precise source of primary gasp drive in the cat is not known (70–72), we speculate that this onset is due in part to the accumulation of neuromodulators, including 5-HT and substance P released by prior and ongoing raphe activity (29), altered inhibition (73), and factors released by local hypoxia-sensing glia (41, 62, 74, 75). The present results suggest that burst efficacy is further amplified by downstream recurrent excitation and disinhibition of inspiration within microcircuits of the inspiratory neuron chain; evidence for disinhibitory modulation of inspiratory drive by changes in blood pressure has been reported in previous studies (39). Efference copies (i.e., corollary discharge such as that which occurs with axon collaterals) of the inspiratory drive engage raphe and pontine circuits in a distributed network of coordinated gasp-related bursting (Fig. 6, *right*, dashed lines). Although the specific functions of the coordinated gasp-synchronous bursting detected in the raphe and pons remain unknown, continued burst-synchronous neuromodulator release by raphe neurons may serve to further enhance target neurons at multiple brain stem and spinal sites (Fig. 6, *right*, wavy lines), thereby evoking additional amplifying recurrent circuit loops and the transition from the early low gasp drive state to the later high gasp drive state (e.g., Fig. 4 and Fig. 5), all of which can potentially contribute to successful autoresuscitation.

### Limitations and Consideration of Methods and Approach

Parallel spike train recordings from multiple sites within the respiratory brain stem permitted the detection of correlational signatures of neuronal connectivity. Cross-correlation analysis has been shown to be sufficiently sensitive to detect evidence for functional interactions among brain stem respiratory neurons (37, 76–79). An offset peak or trough in a cross-correlation histogram is commonly interpreted as a sign of functional excitation or inhibition, respectively (80, 81). These features, together with the firing rate modulations associated with the different respiratory motor patterns, suggested several new hypotheses on circuit operations that contribute to the generation of the hypoxia-evoked motor patterns. Advantages and limitations of these approaches have been published (34, 42, 82, 83).

We note several caveats regarding our approach. Some brain stem regions with neurons known to be modulated by hypoxia were not monitored in this study, including the retrotrapezoid nucleus and parafacial respiratory group (RTN-pF) and downstream autonomic circuits (84). Although neurons in the region of the RTN-pF were not monitored in this study, prior work identified evidence for inspiratory drive projections to that region and reciprocal interactions targeting VRC microcircuits, indicating a network architecture with multiple routes appropriate for gain modulation of breathing by central and peripheral chemoreceptors (35, 36, 38, 45).

There may be other regions that are important in gasp generation as well. Additionally, the mechanisms for gasp generation we have identified and hypothesized in the present study may not be inclusive. We have described an all hands on deck process for autoresuscitative gasping, and this by necessity would require a variety of mechanisms reflecting the coordinated effort of a distributed network, including autonomic components. The broader conclusions we draw are not limited to the data presented here. For example, the circuits illustrated in Fig. 6 demonstrating normoxia and hypoxia are informed not only by the findings of the present study but also by a large body of previous work by our group and others (4, 35, 85).

The cat expresses a wide repertoire of respiratory-related behaviors relevant to human health and is an established in vivo model system for studying the mammalian respiratory brain stem (76, 85–87). Decerebration avoids the effects of anesthetics; chemoreceptor-evoked ventilatory responses to hypoxia are similar to those observed in cats that are awake (88). Our techniques allow us not only to record many neurons at one time but to detect a variety of functional interactions as well. Themes detected in more than one animal and within more than one dataset provide a basis for further hypotheses and investigation.

In the present study, gasping did not change ventilatory efforts because artificial ventilation was used and the cats were neuromuscularly blocked. To evaluate the short-term consequences of improved gas exchange on the respiratory motor pattern, air was reintroduced into the ventilator and blood pressure was supported to maintain adequate perfusion pressure to the brain and other organ systems. We did not dissociate the extent to which a reduction in blood pressure per se contributed to altered network activity and associated changes in blood pressure, either through reduced brain stem perfusion or altered baroreceptor modulation of the respiratory network (31, 39, 89, 90).

### Conclusions

We report that hypoxia-evoked gasps are amplified through a disinhibitory microcircuit within the inspiratory neuron chain and a distributed efference copy mechanism to generate concurrent gasp-synchronous discharges in the raphe-pontomedullary respiratory network. The present results and a large body of prior work are consistent with the idea that brain stem circuits are engaged during the sequential progression of behaviors in response to hypoxia, from the onset of augmentation to apnea and autoresuscitative gasping. Resulting large tidal volumes and altered intrathoracic pressure can enhance blood flow and sympathetic activity. Collectively, these processes operate to bootstrap cardiovascular function and enhance perfusion of carotid body chemoreceptors and their central actions on the brain’s cardiorespiratory control network, and potentially facilitate resuscitation and recovery during cardiac arrest (1, 11) or seizures (2, 91). The responses of neurons to hypoxia, their functional connections, efference copy of gasp drive, and coordinated step increases in blood pressure support a distributed brain stem network model for enhancing and expanding inspiratory drive during gasping that begins with disinhibition by expiratory neurons and initial decrease in inspiratory drive.

## DATA AVAILABILITY

Data will be made available upon reasonable request.

## GRANTS

This work was supported by NIH Grants HL63175 (to I. C. Solomon), R01/37 NS19814 (to B. G. Lindsey), HL163008 (to D. C. Bolser), HL155721 (to D. C. Bolser), NS46062 (to B. G. Lindsey) as part of the NSF/NIH Collaborative Research in Computational Neuroscience (CRCNS) Program, and Common Fund Award OT2OD023854 (to D. C. Bolser).

## DISCLOSURES

No conflicts of interest, financial or otherwise, are declared by the authors.

## AUTHOR CONTRIBUTIONS

S.C.N., I.C.S., K.F.M., and B.G.L. conceived and designed research; S.C.N., I.C.S., K.F.M., and B.G.L. performed experiments; S.C.N., L.S.S., K.E.I., R.O., P.A.V., D.S., D.C.B., K.F.M., and B.G.L. analyzed data; S.C.N., L.S.S., K.E.I., R.O., J.B.D., D.C.B., K.F.M., and B.G.L. interpreted results of experiments; L.S.S., P.A.V., and B.G.L. prepared figures; S.C.N., L.S.S., K.E.I., D.C.B., and B.G.L. drafted manuscript; S.C.N., L.S.S., K.E.I., J.B.D., I.C.S., D.C.B., K.F.M., and B.G.L. edited and revised manuscript; S.C.N., L.S.S., K.E.I., R.O., J.B.D., P.A.V., D.S., I.C.S., D.C.B., K.F.M., and B.G.L. approved final version of manuscript.
